# Open access and beyond

**DOI:** 10.1186/1476-4598-5-35

**Published:** 2006-09-06

**Authors:** Shawn Mathur, Christian Schmidt, Chhaya Das, Philip W Tucker

**Affiliations:** 1 Section of Molecular Genetics and Microbiology and Institute for Cellular and Molecular Biology, University of Texas, 1 University Station, A 5000, Austin, TX 78712, USA; 2 Molecular Cancer, BioMed Central Ltd, Middlesex House, 34-42 Cleveland Street, London W1T 4LB, UK

## Abstract

Uncensored exchange of scientific results hastens progress. Open Access does not stop at the removal of price and permission barriers; still, censorship and reading disabilities, to name a few, hamper access to information. Here, we invite the scientific community and the public to discuss new methods to distribute, store and manage literature in order to achieve unfettered access to literature.

## 

Scientists must be knowledgeable about recent developments in their areas of research. A good scientist reads publications and melds published evidence and theories with his or her own ideas and hypotheses to create refined experiments. The resulting proposal is then submitted to a granting agency. A decision is made about the quality of the proposal, and previously published articles are used as a benchmark of productivity and a predictor of future success. If a project is funded, experiments are then finalized, performed, analyzed, and discussed in collaboration with other laboratories. These observations, in turn, are shared more broadly with the research community via published articles that are quality controlled by the peer-review process. The accepted publication may lead to additional theories or, in rare cases, the complete understanding of a given problem.

Does everybody have unfettered access to all information? The disappointing answer is no.

Special interests, such as securing a monetary and/or intellectual advantage or even keeping a dictatorial government in power, may be sufficient enough to deprive a population of information. In this context, it remains to be debated whether barriers, by virtue of setting up limitations and providing high-level rewards for overcoming, are powerful stimuli for progress.

Considerable progress has been made, however, regarding the distribution and availability of information. Development of the means to deliver ideas and published research findings – language barriers not considered – are as important as their distribution, storage and management. With connectivity to the Internet, nearly universal access to information is at hand. Unfortunately, censorship and connectivity barriers persist as powerful impediments that restrict access to data [1].

In the case of scholarly communication, libraries have neither the capacity to store the physical content of all printed articles nor the budget to afford rising subscription prices for the growing number of journals [1-3]. As an alternative, Open Access removes price and permission barriers [3].

Open Access does not stop at this. Remaining barriers, such as censorship and language, need to be removed in order to fulfill the Open Access definition [1], opening the horizon for the 'Open Research Web' [4].

As it stands, there are two routes to Open Access, namely green (Green OA) and gold (Gold OA), as introduced in [5]. Under the Gold OA Route, articles are published as Open Access articles and the submitting author and/or the author's institution cover the article processing fee [5]. The Gold Open Access Route is preferred by the vast majority of Open Access publishing houses, the two most prestigious publishers are cited here [6,7].

Under Green OA, the author self-archives the article at the time of acceptance, according to the journal's embargo policy, if applicable [5]. This embargo policy prevents free and immediate access to the information. Therefore, Green OA is not in agreement with the Bethesda Statement [8] and, consequently, not an appropriate term to use. Since there is no action without a purpose, this confusing usage of terms plays into the hands of toll-access publishers. The terms 'Green Open Access' and 'Gold Open Access' are created to confuse the public and weaken the Open Access movement. We strongly suggest and encourage the usage of the term **Open Access **for publication and archiving activities that are in perfect agreement with the Bethesda statement [8]. Unfettered access to literature is Open Access.

BioMed Central, the publisher of *Molecular Cancer*, is a publishing house committed to Open Access publishing and archiving according to the Bethesda Statement [8] and as outlined in the BioMed Central Open Access Charter [7].

Scientific journals set their own scope as to what kinds of articles to publish. For instance, PloS Biology [9] and BMC Biology [10], two Open Access journals, aim to be the home for the most fascinating studies in life science. Other journals, such as Nucleic Acids Research [11] and The Journal of Insect Science [12], are also Open Access but have a different scope. It is obvious that all journals live within, and are supported by, the scientific community. Fellow colleagues edit the journals and recruit outstanding scholars to editorial boards. In turn, the journal receives submissions and publishes selected articles. In that sense, journals form communities.

Inevitably, some journals, even those within one publishing house, have overlapping scopes and form a close association, independent as to whether editors transfer reviews to another journal's editors. Examples may be the family of PLoS journals and BMC-series journals.

The speed of scientific progress almost requires scientists to specialize their efforts. Ergo, the most cutting edge science is published in 'community' journals and major break through findings find their home in journals aiming for more general articles. In that context, Open Access benefits the scientific community [3]. For life science, PubMed Central (PMC) [13] must be the repository for all articles because of its connection to all the other NCBI databases [14]. The result will be a truly connected archive.

So far, there is no equivalent of PMC for other disciplines, such as physics, chemistry or mathematics. Given the case that all NCBI archives become connected and searches between databases become possible, the Open Research Web [4] will be at hand.

In conclusion, there is much to do to achieve Open Access in its true meaning. Barriers have to be removed and technology has to be advanced. The scientific community and the public need to define areas of immediate concern and long-term goals.

We, therefore, open the discussion about Open Access and beyond.

## Competing interests

S.M., C.D. and P.W.T. declare that there are no competing interests. C.S. is Deputy Editor of *Molecular Cancer *and receives no remuneration for his efforts.

## Authors' contributions

C.S. wrote and finalized this manuscript. S.M., C.D. and P.W.T. provided critique and suggestions. All authors read and approved the final manuscript.
